# CRISPR/Cas9-mediated enhancement of semi-dwarf glutinous traits in elite Xiangdaowan rice (*Oryza sativa* L.): targeting *SD1* and *Wx* genes for yield and quality improvement

**DOI:** 10.3389/fpls.2024.1333191

**Published:** 2024-02-16

**Authors:** Quanxiu Wang, Haolin Gao, Ke Liu, Honglin Wang, Fan Zhang, Lanmeng Wei, Kaijing Lu, Mengmeng Li, Yiming Shi, Jinhui Zhao, Wei Zhou, Bo Peng, Hongyu Yuan

**Affiliations:** College of Life Sciences, Institute for Conservation and Utilization of Agro-Bioresources in Dabie Mountains, Xinyang Normal University, Xinyang, China

**Keywords:** rice, Sd1, Wx, gibberellin, amylose, transcriptome

## Abstract

In rice cultivation, the traits of semi-dwarfism and glutinous texture are pivotal for optimizing yield potential and grain quality, respectively. Xiangdaowan (XDW) rice, renowned for its exceptional aromatic properties, has faced challenges due to its tall stature and high amylose content, resulting in poor lodging resistance and suboptimal culinary attributes. To address these issues, we employed CRISPR/Cas9 technology to precisely edit the *SD1* and *Wx* genes in XDW rice, leading to the development of stable genetically homozygous lines with desired semi-dwarf and glutinous characteristics. The *sd1-wx* mutant lines exhibited reduced gibberellin content, plant height, and amylose content, while maintaining hardly changed germination rate and other key agronomic traits. Importantly, our study demonstrated that exogenous GA_3_ application effectively promoted growth by compensating for the deficiency of endogenous gibberellin. Based on this, a semi-dwarf glutinous elite rice (*Oryza sativa* L.) Lines was developed without too much effect on most agronomic traits. Furthermore, a comparative transcriptome analysis unveiled that differentially expressed genes (DEGs) were primarily associated with the anchored component of the membrane, hydrogen peroxide catabolic process, peroxidase activity, terpene synthase activity, and apoplast. Additionally, terpene synthase genes involved in catalyzing the biosynthesis of diterpenoids to gibberellins were enriched and significantly down-regulated. This comprehensive study provides an efficient method for simultaneously enhancing rice plant height and quality, paving the way for the development of lodging-resistant and high-quality rice varieties.

## Introduction

Rice (*Oryza sativa* L.) sustains over half of the world’s population as a staple food, making the enhancement of grain quality and yield a global priority (Chandler and Wessler, 2001; Khush, 2005; Zhang, 2007; Demont and Stein, 2013; Qiao et al., 2021). Xiangdaowan (XDW) rice, esteemed for its robust aromatic qualities, confronts a challenge with its traditional varieties exhibiting excessive plant height and poor lodging resistance, adversely impacting rice yield (Singh et al., 2011). Conversely, excessively short plants produce smaller grains and exhibit diminished disease resistance, emphasizing the critical importance of maintaining an optimal plant height for maximizing rice yield (Liu et al., 2018). Gibberellin (GA), a pivotal plant hormone governing stem elongation, holds a key role in regulating plant height (Zhu et al., 2006; Zhang et al., 2014). Numerous genes contribute to GA biosynthesis and signaling, with *SD1* (semi dwarf1) standing out as the “Green Revolution” gene. *SD1* encodes the crucial enzyme gibberellin 20-oxidase 2 (GA20ox2), essential for gibberellin synthesis (Spielmeyer et al., 2002). Mutations in *SD1* result in reduced gibberellin production, leading to a shorter plant stature (Monna et al., 2002; Sasaki et al., 2002). Since the 1930s, the *sd1* allele has been used in rice breeding to produce rice varieties with short stature and excellent lodging resistance (Sakamoto and Matsuoka, 2004; Asano et al., 2007). The tall plant phenotype is governed by the *SD1* allele, while the recessive *sd1* allele induces the semi-dwarf phenotype (Monna et al., 2002; Berry et al., 2004; Magome et al., 2013). Gibberellins, such as GA_1_ and GA_4_, composed of diterpene carboxylic acids, play crucial roles in plant growth and development especially in regulating plant height (Thomas and Hedden, 2006; Hedden and Thomas, 2012; Marciniak et al., 2018).

Amylose content (AC) plays a pivotal role in the culinary and physical attributes of rice, exerting a profound influence on its eating and cooking quality (ECQ) (Li et al., 2016; Huang et al., 2021; Zhang et al., 2021a, b). The *Waxy* gene (*Wx*), a key player in this saga, encodes granule-bound starch synthase (GBSS) and stands as a major architect dictating amylose synthesis in rice grains. This synthesis, in turn, modulates the AC, gel consistency (GC), and viscosity (Wang et al., 1990; Su et al., 2011; Zhou et al., 2021; Zhang et al., 2021a). Rice varieties are classified into five types based on their AC: high (>25%), intermediate (20-25%), low (10-19%), very low (3-9%), and glutinous (0-2%) (Li and Gilbert, 2018). To date, at least nine allelic variations of *Wx*, *Wx^la^/Wx^mw^*, *Wx^lv^*, *Wx^a^*, *Wx^b^*, *Wx^in^*, *Wx^op^/Wx^h^* ^p^, *Wx^mp^*, *Wx^mq^*, and *wx*, have been related to the five AC types found in rice cultivars (Ayres et al., 1997; Cai et al., 1998; Sato et al., 2002; Larkin and Park, 2003; Wanchana et al., 2003; Mikami et al., 2008; Liu et al., 2009; Yang et al., 2013; Zhang et al., 2019; Zhou et al., 2021; Zhang et al., 2021a). In general, rice varieties with higher *Wx* gene expression exhibit higher GBSS enzyme activity, resulting in a higher AC content and, conversely, lower AC content (Kumar and Khush, 1986; Su et al., 2011). Researchers have explored manipulating the promoter or exon region of the *Wx* gene to regulated AC, resulting in the development of soft or glutinous rice lines (Han et al., 2018; Yang et al., 2018; Zhang et al., 2018; Pérez et al., 2019; Yunyan et al., 2019; Zeng et al., 2020; Huang et al., 2021; Yang et al., 2022; Tian et al., 2023; Zhang et al., 2023).

Both *Wx* and *SD1* genes play crucial roles in determining rice grain quality and yield potential, respectively. Despite breeding efforts using diverse strategies such as marker-assisted selection, progress has been sluggish, especially in enhancing complex and multiple traits. In recent years, CRISPR/Cas9 genome editing technology has been successfully applied to improve crop traits in rice, maize, wheat, sorghum and soybean, which offers rapid and efficient trait modification (Jiang et al., 2013; Mao et al., 2013; Shan et al., 2013; Liang et al., 2014; Wang et al., 2014; Li et al., 2015). Although some researchers have employed gene editing technology to improve plant height or quality by targeting the *SD1* or *Wx* gene in elite rice landrace or hybrid rice (Zhang et al., 2018; Han et al., 2019; Hu et al., 2019; Yunyan et al., 2019; Nawaz et al., 2020; Zeng et al., 2020; Yang et al., 2022), the simultaneous editing of both genes in the same rice variety remains uncharted.

In this study, we examined Xiangdaowan (XDW) rice, a fragrant, high-quality rice variety characterized by poor lodging resistance and tough rice texture. Despite its robust aromatic qualities, it suffers from a tall stature and elevated AC, resulting in a low harvest index and suboptimal rice quality. Enhancing lodging resistance and refining the overall quality of rice are crucial objectives that can augment yield and the breeding and utilization value by altering plant height and AC. To address these challenges, we utilized CRISPR/Cas9 technology to develop *sd1-wx* double mutants in Xiangdaowan (XDW) rice. Across various generations, we successfully obtained genetically stable, homozygous mutants. Subsequently, we investigated the phenotype of *sd1-wx* double mutants, and constructed semi-dwarf glutinous rice varieties with low plant height and AC content without disrupting other essential agronomic characteristics. Further unraveling the intricacies of the *SD1* and *Wx* genes, we conducted comparative RNA-seq analysis on *sd1-wx* double mutants and the wild type (WT). In summary, our study not only promises increased yield and the provision of high-quality resources for the improvement of new rice varieties but also sheds light on the functional roles of *SD1* and *Wx* genes through advanced RNA-seq analysis.

## Materials and methods

### Plant material and measurement of agronomic traits

The genetic groundwork for the transgenic plants comprises the esteemed rice landraces Xiangdaowan (*Oryza sativa* L., ssp. Japonica). All rice cultivation occurred in the natural settings of paddy fields at Xinyang Normal University in Xinyang, China (32°8’30’’N, 114°2’8’’E). Following to rice harvest, a minimum of 10 randomly selected rice plants underwent analysis for agronomic traits, including plant height, tiller number, grain weight, and grain number per panicle.

### Construction of CRISPR/Cas9 vectors and plant transformation

The CRISPR/Cas9 vector system tailored for simultaneous targeting of multiple gene sites in monocot plants was procured from Biogle Biotechnology Co., Ltd. The design of gRNA target sequences aligned with the exon sequence of *SD1* (LOC_Os01g66100) and *Wx* (LOC_Os06g04200), as outlined by the Rice Genomics Annotation website (http://rice.plantbiology.msu.edu/). In a nutshell, these target site sequences were incorporated into the sgRNA expression cassette, which included a rice promoter, and subsequently integrated into the *Bsa* I restriction sites of the pCRISPR/Cas9-BGK032 vector. Detailed primer information for constructing the sg RNA vectors for *Wx* and *SD1* can be found in **Supplementary Table S1**. Following this, independent introductions of CRISPR/Cas9 constructs were made into *Agrobacterium tumefaciens* strain *EHA105*, followed by transformation into the elite rice landraces Xiangdaowan using a method described previously (Nishimura et al., 2006). *Hygromycin* phosphotransferase (*hpt*) served as the plant selectable agent during the screening process for positive rice transformants.

### RNA-seq analysis

To assess the transcriptome profiling of edited lines resulting from the *Wx* and *SD1* gene modifications, we conducted transcriptomic analysis using both wild-type (WT) and mutant plants. Stem tissues at the heading stage were utilized for RNA extraction. The RNA samples underwent sequencing by APTBIO Co. in Shanghai, China, utilizing the Illumina HiSeq™ 2000 platform. For robustness, three biological replicates were included in the transcriptomic analysis. The analytical methods for transcriptomics were based on previous method (Wang et al., 2023). The resulting clean reads, stored in FASTQ format, were mapped to the reference genome using Bowtie2. Alignment to the Nipponbare reference genome (http://rice.plantbiology.msu.edu/) was achieved with an ideal match using HISAT2 software. The expression level of each gene was normalized to fragments per kilo base per million (FPKM) mapped reads. Differentially expressed genes (DEGs) between mutant and WT plants were identified based on a fold change ≥ 2 and a *p*-value < 0.05. Mapping of DEGs to GO terms was performed, and the count of genes for each term was determined. Significance of GO term enrichment was assessed using the hypergeometric test, with *p*-values adjusted. GO terms were considered significantly enriched only when both the *p*-value and *p*-adjusted value were < 0.05. Additionally, KEGG analysis was employed to identify significantly enriched signaling and metabolic pathways for each DEG compared with the reference genome. KEGG terms with both a *p*-value and *p*-adjusted value of < 0.05 were considered significantly enriched.

### Real-time quantitative PCR assays

In order to verify the result of RNA-seq result, RT-qPCR was carried out on selected genes. Trizol reagent was used to isolate total RNA from rice stems tissue by following the manufacturer’s protocol. And then 3 µg RNA samples were synthesized to cDNA using the reverse transcriptase. Quantitative RT-PCR assays were conducted analysis on the CFX96 thermocycler (Bio-rad. USA) PCR System using the SYBR green master. We used three biological and technical replicates. The rice ubiquitin gene was used as an internal control. The expression level of each gene was determined based on previous method (Livak and Schmittgen, 2001). Primers for real-time quantitative PCR are shown in **Supplementary Table S1**.

### Seed germination rate measurement and plant hormone treatments

Following harvest, rice was subjected to a drying process at 37°C for 5 days, after which it was immersed in water, and then placed in a growth chamber maintained at 30 °C in darkness. The assessment of seed dormancy involved visual scoring, specifically when the radicle exceeded 3 mm in length. For H_2_O and 4 µM GA_3_ treatments, approximately 50 well-developed seeds were evenly distributed on wet filter paper in culture dishes with a diameter of 90 cm and were subsequently treated with the 8 mL specified reagents (H_2_O or 4 µM GA_3_) in incubator at 30°C. It was ensured that the seeds remained moist during this experiment. Germination rates were monitored daily from day 3 to day 5. This process was replicated three times. The resulting germination percentages were averaged for subsequent genetic analysis.

### Quantification of the endogenous plant hormone

Guangzhou Hellogene Biotechnology Company conducted the detection of GA compounds using the following procedure: The samples (~1 g) were finely powdered and combined with 4 mL of acetonitrile (v/v) containing 20 ng of each deuterium-labeled internal standard ([^2^H_2_]GA_3_ and [^2^H_6_]ABA). This mixture was then placed on a shaker at 300 rpm in the dark at 4 °C for 24 hours. Afterward, the samples underwent centrifugation (12,000 g, 5 minutes), and the resulting supernatant was carefully transferred to a clean tube. To further purify the extract, it was passed through a poroshell 120 SB-C18 column, pre-equilibrated with methanol, followed by an extraction buffer. The column was subsequently rinsed with 500 μL of methanol acidified with 0.1% methanoic acid. The purified extract was then dried using nitrogen gas and reconstituted with 400 μL of 100% methanol at 4 °C in the dark for 24 hours. After centrifugation (12,000 g, 5 minutes), the supernatant was transferred into HPLC vials and subjected to liquid chromatography-mass spectrometry (LC-MS) analysis using a SCIEX6500QTRAP LC/MS/MS system, equipped with an ESI Turbo Ion-Spray interface.

### Measurement of grain physicochemical properties

After plant maturation, the harvested rice grains underwent air-drying and were subsequently stored at room temperature for a minimum of three months prior to testing. The flours and starches from brown rice were prepared as previously described (Bao et al., 2006). Measurement of the apparent amylose content (AC) and gel consistency (GC) of brown rice flours was conducted according to the method described by Bao et al (Bao et al., 2006). For scanning and transmission electron microscopy analyses of starch granules, cross sections of brown white rice grains and rice powders were gold-coated under vacuum. Starch granule morphology was then examined using a scanning electron microscope (Regulus-8220, Japan) at an accelerating voltage of 3.0 kV and magnifications of 400×, 2,500×, and 3,000×. The SEM analysis was based on at least three biological replications of mounted specimens, and all procedures strictly adhered to the manufacturer’s protocol.

The GT (gelatinization temperature) values were estimated indirectly by the alkali spreading score (ASS) method. Briefly, 6 grains of whole milled white rice from each strain were placed in a 90 mm petri dish along with 20 ml of 1.7% potassium hydroxide solution. The samples were separated from each other using forceps and incubated at 30°C for 23 h to allow the rice grains to spread. The spreading score of the grains was recorded visually follow previous method. The ASS score of 1-7 was recorded depending on the appearance and degree of dispersion of the endosperm. Unaffected or slightly swollen endosperm was recorded as 1, while completely disappeared was recorded as 7. The GT value was inversely proportional to the ASS score. There are three grades of GT: ASS grades 1 to 2 are high GT means gelatinization temperature >75°C, grades 3 are high-intermediate GT means gelatinization temperature between 70°C-74°C, grades 4 to 5 are intermediate GT means gelatinization temperature between 66°C-69°C, and grades 6 to 7 are low GT means gelatinization temperature between 55°C-65°C.

### Statistical analysis

To characterize the samples, we conducted measurements in triplicate, unless stated otherwise. All data are expressed as mean ± standard deviation (SD). Statistical analyses, including one-way ANOVA and Student’s t-test, were performed using the SPSS 16.0 software. *P*<0.05 was deemed statistically significant.

## Results

### Editing of *OsSD1* and *OsWx* genes via CRISPR/Cas9 technology

**Figure 1:**
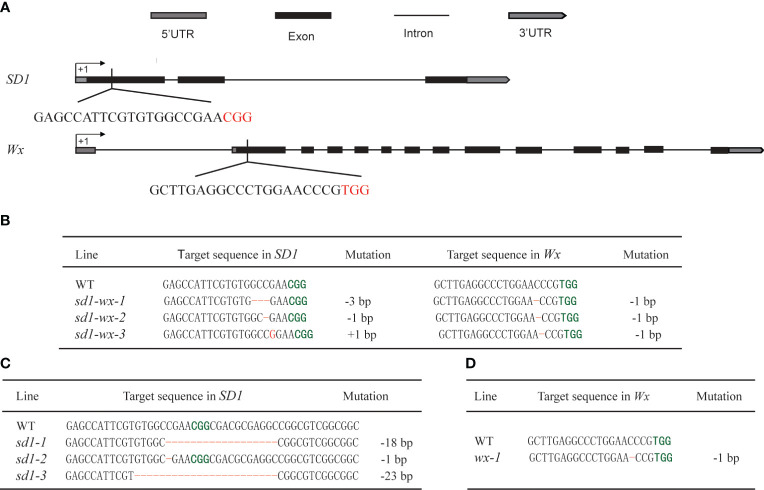
Targeted editing of *SD1* and *Wx* genes based on the CRISPR/Cas9 system. **(A)** Schematic representation of the gene structure and target sites of *SD1* and *Wx* genes. Introns and exons are indicated by black lines and black rectangles, respectively, and PAM sequences are highlighted in red. **(B)** The *sd1* and *wx* double mutations in the edited T_1_ lines. **(C)** The *sd1* single mutation in the edited T_1_ lines. **(D)** The *wx* single mutation in the edited T_1_ lines. Deletions and insertions are highlighted in red and the PAM sequences are highlighted in green, respectively.

To enhance the plant height and quality of elite rice landrace Xiangdaowan (XDW), we employed CRISPR/Cas9 technology to create single mutants for the *sd1* and *wx* genes, as well as double mutants (*sd1-wx*). Briefly, we designed and constructed the pCRISPR/Cas9-BGK032-*SD1* and pCRISPR/Cas9-BGK032-*Wx* single target vectors, along with the pCXUN-*Wx-SD1* dual target vector (**Figure 1A**). These vectors were introduced into XDW callus through *Agrobacterium*-mediated transformation. We obtained 21 XDW*^sd1^* T_0_ transgenic plants, 5 XDW*^wx^* T_0_ transgenic plants and 20 XDW*^sd1-wx^* T_0_ double mutants transgenic plants, respectively. Subsequently, we utilized PCR to select plants that were free of *Cas9* and *HPT* plants from the transgenic (T_1_–T_2_) segregating families. Sanger sequencing of *SD1* and *Wx* target sites identified various insertion/deletion mutations. To produce desirable homozygous and non-transgenic plants, we closely monitored the genotypes of the target sites as well as the segregation of T-DNA from T_1_ progeny plants to T_3_ progeny plants. Ultimately, we successfully obtained three lines of homozygous and T-DNA-free editing in the single mutant XDW*^sd1^*, one in the single mutant XDW*^wx^*, and three in the double mutants XDW*^sd1-wx^*. XDW*^sd1-^* ^1^ contained eighteen nucleotide deletion in *SD1*, XDW*^sd1-^* ^2^ consisted of one nucleotide deletion (ΔC) in *SD1*, and XDW*^sd1-^* ^3^ contained twenty-three nucleotide deletion in *SD1* (**Figure 1C**). XDW*^wx^* ^-1^ contained one nucleotide deletions (ΔC) in *Wx* (**Figure 1D**). XDW*^sd1-wx^* ^-1^ comprised of three nucleotide deletion (ΔGCC) in *SD1* and one nucleotide deletions (ΔC) in *Wx* (**Figure 1B**). XDW*^sd1-wx^* ^-2^ consisted of one nucleotide deletion (ΔC) in *SD1* and *Wx* (**Figure 1B**), respectively. XDW*^sd1-wx^* ^-3^ contained one nucleotide insertion (ΔG) in *SD1* and one nucleotide deletion (ΔC) in *Wx* (**Figure 1B**). Most of the mutations were predicted to cause frameshifts, resulting in the translation of non-functional truncated proteins. In conclusion, we employed CRISPR/Cas9 technology to precisely edit the *SD1* and *Wx* genes in XDW rice, leading to the development of stable genetically homozygous lines.

### Endogenous GA concentrations and plant heights in *sd1* mutants

**Figure 2:**
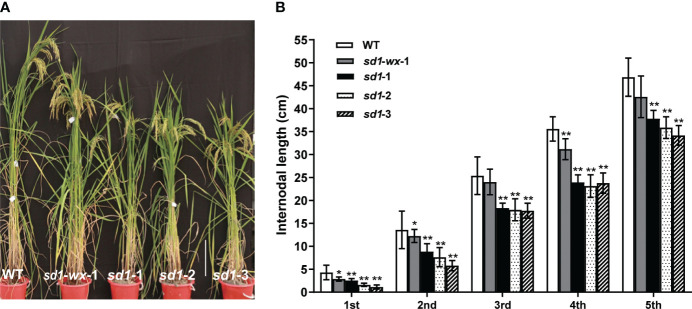
Phenotypes of *sd1* and WT lines. **(A)** The plant height between mutant lines and WT lines at heading stage. Bar = 25 cm. **(B)** Internode length of mutant and WT lines. **indicates the significant difference (*p* < 0.01).

To evaluate the effect of rice plant height in *sd1* mutants, we systematically measured the plant height and stem internode length of transgenic plants. The *sd1* mutant lines had shorter plant heights and lower GA concentrations compared to WT plants. Specifically, the mutant lines XDW*^sd1-wx^* ^-1^ XDW*^sd1-^* ^1^, XDW*^sd1-^* ^2^, and XDW*^sd1-^* ^3^ showed plant heights of 136.9 cm, 117.6 cm, 114.9 cm and 105.7 cm in the T_2_ generation, respectively, whereas WT had a maximum height of 158.8 cm (**Table 1**). Analysis of the length of each stem internode showed that the reduction in plant height was caused by a different degree of decline in the length of each stem internode (**Figure 2**). In order to confirm whether the reduction of mutant lines plant height was due to the reduction of endogenous gibberellin content, we measured the content of gibberellin (GA_1_, GA_3_, GA_4_) through LC-MS analysis. The mutant line XDW*^sd1-^* ^3^, with the minimum plant height, had the lowest GA_3_ content. The mutant lines XDW*^sd1-^* ^1^ and XDW*^sd1-^* ^2^ had the lowest GA_1_ and GA_4_ content (**Table 1**), respectively. In contrast, the WT with the maximum height had the highest gibberellin content. This suggests that editing the *SD1* gene significantly reduces plant height by decreasing endogenous gibberellin levels.

**Table 1:** GA content (µg/kg FW) and PH (cm) in WT and mutant lines.

Line	GA_1_	GA_3_	GA_4_	PH
WT	1.073 ± 0.058	0.655 ± 0.025	0.324 ± 0.005	158.8 ± 4.2
*sd1-wx-*1	0.841 ± 0.054^**^	0.501 ± 0.020^**^	0.265 ± 0.006^**^	136.9 ± 4.8^**^
*sd1-*1	0.611 ± 0.010^**^	0.426 ± 0.025^**^	0.202 ± 0.006^**^	117.6 ± 4.6^**^
*sd1-*2	0.729 ± 0.026^**^	0.319 ± 0.021^**^	0.158 ± 0.002^**^	114.9 ± 2.9^**^
*sd1-*3	0.657 ± 0.009^**^	0.281 ± 0.011^**^	0.177 ± 0.002^**^	105.7 ± 4.3^**^
*wx-*1	*-*	*-*	*-*	157.3 ± 4.0 ^ns^

The data are the means of three replicates. ^ns^ and ^**^represent non-significant differences and the significant (p<0.01), respectively.

### Seed germination and effect of exogenous GA_3_ in *sd1* mutants

**Figure 3:**
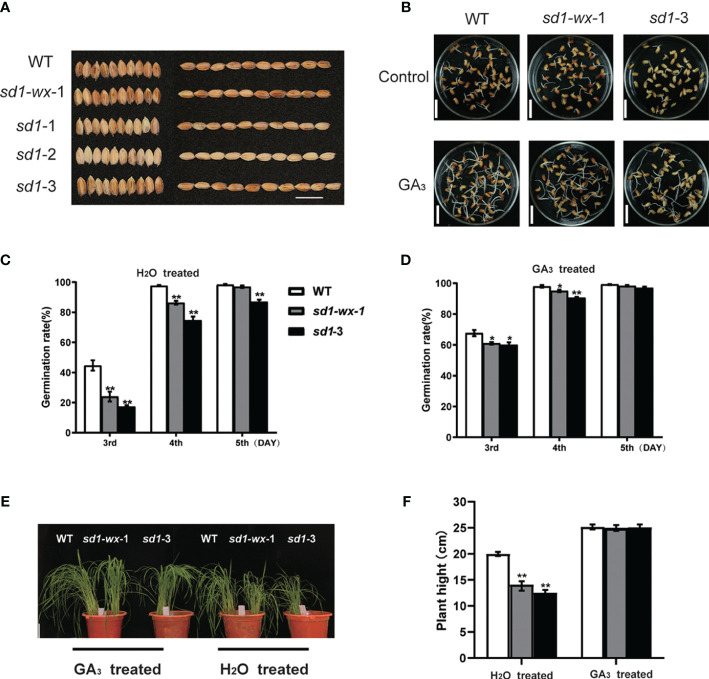
Seed germination and effect of exogenous GA_3_ in mutant lines and WT. **(A)** The grain phenotype of mutant line and WT; Bar = 15 mm. **(B)** Germination phenotypes of WT, *sd1-wx-*1, and *sd1-*3. The germination phenotypes are shown at the 3th day after imbibition. Bar = 15 mm. The germination rates of WT, *sd1-wx-*1, and *sd1-*3 under 4 µM GA_3_ treated **(C)** and H_2_O treated **(D)**. Seed germination was measured after soaking for 2 days in treatment solution and then imbibing for 3 days. We recorded germination rates from day 3 to day 5. **(E)** Seedlings phenotype (30 day) of WT and *sd1-*3 and *sd1-wx-1* under 10 µM GA_3_ treated and H_2_O treated, respectively. Bar = 4 cm. **(F)** The plant height of WT and *sd1-wx-1* and *sd1-*3 under 10 µM GA_3_ treated and H_2_O treated, ^ns^ and ^**^ indicates the non-significant and significant difference at *p* < 0.01.

The concentration of endogenous gibberellin (GA) is crucial for the successful germination of seeds, ensuring the continuous life cycle of seed plants. Quality seed germination is indicative of robust seedling establishment and overall plant production. Herein, we examined the germination rates of XDW, XDW*^sd1-wx^* ^-1^ and XDW*^sd1-^* ^3^ seeds between H_2_O and GA_3_ treatment groups. After imbibition for 5 days, we observed distinct germination responses among WT and mutant seeds. On the third day, the *sd1-wx-1* mutant exhibited remarkably lower germination rates compared to WT (**Figure 3B**), but this difference disappeared by the fifth day (**Figures 3C, D**). Compared with WT, *sd1-*3 mutants displayed significantly lower germination rates (**Figures 3C, D**). Recognizing the pivotal role of GA in seed germination regulation, we explored the impact of exogenous 4 µM GA_3_ treatment, revealing a significant increase in germination rates. We found that exogenous GA_3_ treatment can partially compensate lower germination rates in *sd1* mutation compared with WT (**Figures 3C, D**). Further investigation indicated that the *sd1-3* mutation somewhat increased seed dormancy at the early stages of seed germination, aligning with previous findings (Ye et al., 2015). Collectively, our results suggest that the *sd1* mutation may reduce endogenous GA_3_ content, leading to decreased germination rates. However, this deficiency can be partially compensated by the application of exogenous GA.

To assess the potential of exogenous gibberellin application in addressing endogenous deficiency, we treated XDW plants and the height-reduced mutant line XDW*^wx-sd1-1^* and XDW*^sd1-^* ^3^ with 10 µM GA_3_ at the seedling stage (30 days old) in the greenhouse. Under controlled conditions, XDW*^wx-sd1-1^* and XDW*^sd1-^* ^3^ plants initially displayed the lower height (14.11 cm, 12.55 cm) compared to WT XDW plants (19.99 cm) (**Figures 3E, F**). Following GA_3_ treatment, the T_2_ mutant line XDW*^wx-sd1-1^* and XDW*^sd1-^* ^3^ exhibited restored plant height (24.95 cm, 25.08 cm), nearly equivalent to that of WT plants (25.17 cm) (**Figures 3E, F**). These findings demonstrate that the application of exogenous GA_3_ can enhance growth by supplementing the deficiency of endogenous gibberellin.

### Development of glutinous rice with decreased amylose content in grains

**Figure 4:**
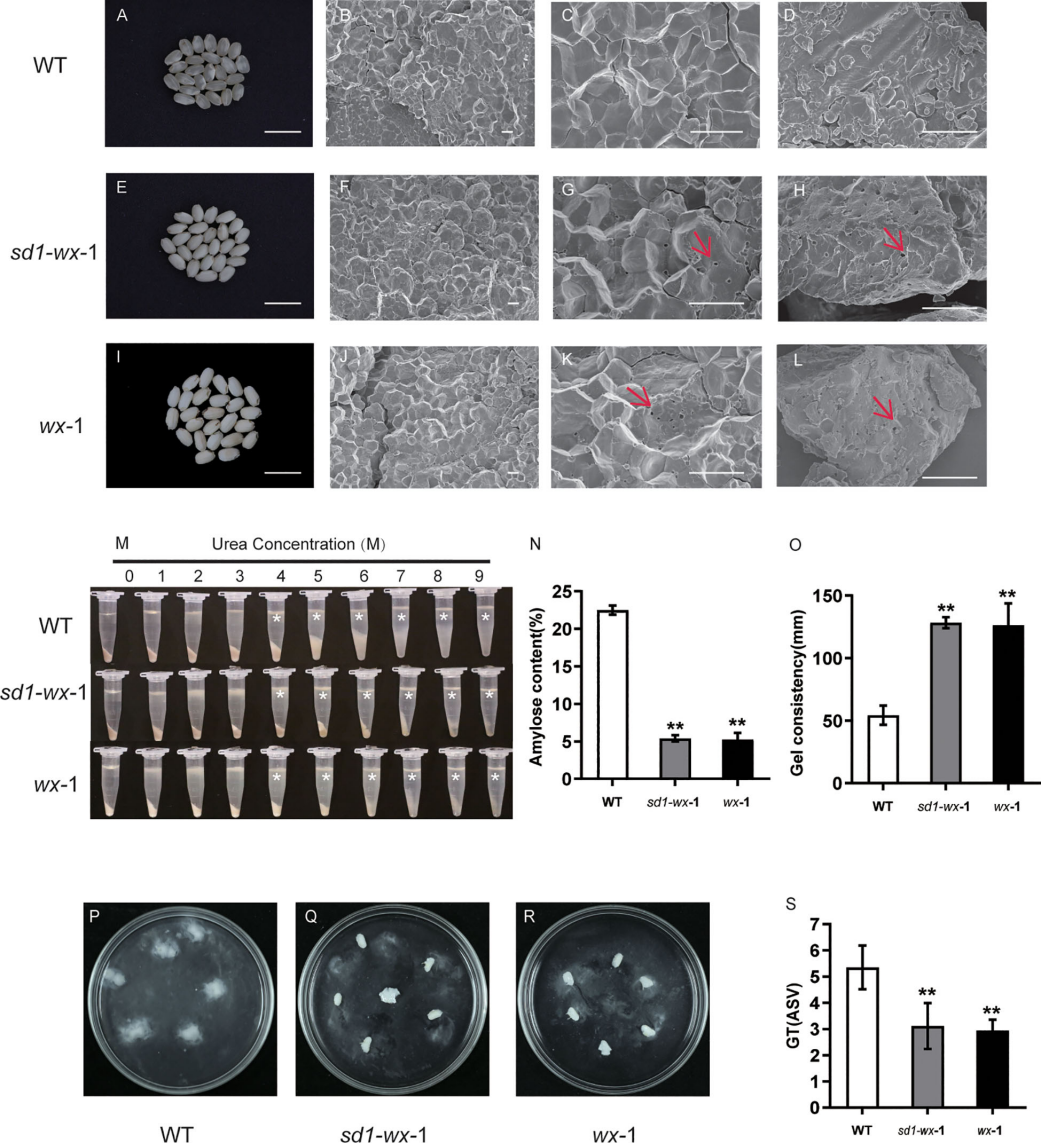
An analysis of starch phenotypic and physicochemical characteristics in WT and *wx* mutants. Comparative analysis of brown rice appearance between WT **(A)** and *sd1-wx*-1 **(E)** and *wx*-1 **(I)**; Bar = 1 cm. Scanning electron microscopy images of cross sections of the WT **(B, C)** and *sd1-wx*-1 mutant **(F, G)** grains and *wx*-1 **(J, K)**. Scale bars, 10 µm and magnifications of 1,000× in **(B, F, J)**; 10 µm and magnifications of 3,000× in **(C, G, K)**. Scanning electron microscope images of WT **(D)** and *sd1-wx*-1 mutant **(H)** and *wx*-1 **(L)** brown rice powders. Scale bars, 10 µm and magnifications of 3,000× in **(D, H, L)**. Small cavities are marked by red arrows. **(M)** Gelatinization properties of starch in urea solutions. Starch powder of WT and *sd1-wx*-1 and *wx*-1 was mixed with different concentrations of urea solution. * indicate the starch from the endosperm of *sd1-wx*-1 and *wx*-1 were more difficult to gelatinize than that from the endosperm of WT in urea solutions of 4-9 M. **(N)** The amylose content in endosperm of WT and *sd1-wx*-1 and *wx*-1. **(O)** The gel consistency in endosperm of WT and *sd1-wx*-1 and *wx*-1. The seed alkali spreading score (ASS) during WT **(P)** and *sd1-wx*-1 **(Q)** and *wx*-1 **(R)**. The GT (gelatinization temperature) values were estimated indirectly by the alkali spreading score (ASS) during WT and *sd1-wx*-1 and *wx*-1 **(S)**. ** indicate statistical significance between WT and *wx* mutant lines at *p*< 0.01.

To determine the grain quality of mutant lines, amylose content (AC) was investigated. The amylose content of the mutant line XDW*^sd1-wx^* ^-1^ and XDW*^wx^* ^-1^ were 5.4% and 5.2%, respectively, which showed a sharp decrease compared with the wild type (**Figure 4N**). All brown rice from the mutant line XDW*^sd1-wx^* ^-1^ and XDW*^wx^* ^-1^ exhibited an opaque appearance (**Figure 4E, I**). In contrast, the WT grains had an AC of 22.5% (**Figure 4N**), and most of the grains displayed a transparent appearance (**Figure 4A**). Alongside the AC, the gel consistency (GC) in the XDW*^sd1-wx^* ^-1^ and XDW*^wx^* ^-1^ mutant line showed a significant increase compared to that of WT (**Figure 4O**). To investigate the microstructural features of mature endosperm, we examined cross sections of the grains using a scanning electron microscope (SEM). The results revealed numerous small cavities in the core of starch granules within the glutinous endosperm of the XDW*^sd1-wx^* ^-1^ and XDW*^wx^* ^-1^ mutant line (**Figures 4F-H, J-L**). Interestingly, similar structures were absent in starch granules from transparent WT grains (**Figures 4B-D**). These small cavities within the starch granules may account for the observed differences in physicochemical properties and transparency between WT and the XDW*^sd1-wx^* ^-1^ and XDW*^wx^* ^-1^ mutant line. The gelatinization characteristics of starch in urea solution can reflect whether the physicochemical properties of rice have changed. Both WT and *wx* mutant line starch powder were dissolved in urea solution with different concentrations (0-9 mol/L). Intriguingly, we observed that at 0-3 mol/L urea concentrations, both starch varieties exhibited a certain resistance to dissolution. However, there were notable changes when the urea concentration elevated to the range of 4-9 mol/L. Starch powder of mutant line XDW*^sd1-wx^* ^-1^ and XDW*^wx^* ^-1^ began to dissolve in a small amount in 4 mol/L urea solution, while the WT was difficult to dissolve (**Figure 4M**). These findings underscore a shift in the physicochemical attributes of starch subsequent to the editing of the *Wx* gene. In order to better evaluate the eating and cooking quality (ECQ), we examined the GT (gelatinization temperature) values by the alkali spreading score (ASS). According to the alkali spreading value, the rice can be categorized into four types according to the GT value: high GT (75-79°C), high-intermediate GT (70-74°C), intermediate GT (66-69°C), and low GT (55-65) °C. In our study, the XDW*^sd1-wx^* ^-1^ and XDW*^wx^* ^-1^ seeds were classified as having high-intermediate GT, while the WT was classified as having low GT (**Figures 4P-S**). Notable alterations include a marked reduction in AC, conspicuous increases in the GC of the gel and the GT, the emergence of small perforations in the endosperm microstructure, and discernible changes in starch gelatinization dynamics when exposed to urea solutions. These results indicate that the edited mutant line XDW*^sd1-wx^* ^-1^ and XDW*^wx^* ^-1^ have the characteristics of glutinous rice with good rice quality.

### Performance of agronomic and quality traits

To evaluate the impact of the *sd1-wx* double mutant lines on yield, we conducted a comprehensive analysis of major agronomic traits across multiple generations, ranging from T_1_ to T_3_ (**Table 2**). Consistently, the results demonstrated discernible effects of the mutations persisting through subsequent generations. Panicle numbers (PN) exhibited no significant difference between the WT plants and mutant lines, indicating a degree of stability in this trait. Significant variations emerged in certain semi-dwarf lines, with shorter panicle length (PL) and lower seed setting rates (SSR). Grain number per panicle (GNPP) displayed no marked distinctions in most mutants when compared to the WT, although specific *sd1-3* mutant lines exhibited diverse effects. Notably, the XDW*^sd1^* ^-3^ mutant line, characterized by minimal plant height, exhibited substantial reductions in PL, GNPP, SSR, 1000-grain weight (GW), and yield per plant (YPP) compared to the WT. Intriguingly, it displayed longer grain length (GL) (**Figure 3A**). Of particular interest is the semi-dwarf XDW*^sd1-wx^* ^-1^ double mutant line, which demonstrated a comparable yield per plant to the WT. Across T_1_ to T_3_ generations, the YPP for WT and XDW*^sd1-wx^* ^-1^ double mutant line exhibited changes from 24.15 g to 25.26 g, 26.32 g to 26.78 g, and 31.97 g to 31.83 g, respectively. Besides, other agronomic traits between WT and XDW*^sd1^* ^-^ *^wx^* ^-1^ double mutant line showed no significant differences. This suggests that XDW*^sd1^* ^-^ *^wx^* ^-1^ double mutant line holds promise as a potential breeding material. The application of CRISPR/Cas9-based mutagenesis targeting *Wx* and *SD1* in generating semi-dwarf glutinous elite rice lines could contribute to enhanced genetic diversity, ultimately benefiting rice yield and quality.

**Table 2:** Major agronomic traits in WT and mutant lines from T_1_ generation to T_3_ generation.

Generation	Line	PN	PL	GNPP	SSR(%)	1000-GW(g)	YPP(g)	GL(mm)	GW(mm)	GT(mm)
T_1_	WT	7.1 ± 1.5	27.38 ± 0.72	122.9 ± 10.3	83.7 ± 4.2	27.88 ± 0.43	24.15 ± 3.81	7.30 ± 0.09	3.60 ± 0.05	2.24 ± 0.07
	*sd1-wx-1*	7.3 ± 1.6 ^ns^	26.04 ± 1.47^*^	126.5 ± 14.3 ^ns^	76.3 ± 3.9^**^	27.29 ± 0.75 ^ns^	25.26 ± 6.50 ^ns^	7.27 ± 0.06 ^ns^	3.59 ± 0.08 ^ns^	2.22 ± 0.08 ^ns^
	*sd1-1*	6.9 ± 0.9 ^ns^	24.46 ± 0.78^**^	126.1 ± 21.1 ^ns^	73.2 ± 4.4^**^	26.12 ± 1.77^*^	22.39 ± 3.96 ^ns^	7.21 ± 0.08^*^	3.48 ± 0.07^**^	2.14 ± 0.08^**^
	*sd1-2*	6.8 ± 0.9 ^ns^	25.15 ± 0.76^**^	123.9 ± 12.4 ^ns^	72.2 ± 5.1^**^	27.06 ± 1.39 ^ns^	22.45 ± 3.32 ^ns^	7.34 ± 0.08 ^ns^	3.64 ± 0.05 ^ns^	2.20 ± 0.09^**^
	*sd1-3*	5.4 ± 1.1^*^	23.10 ± 0.99^**^	97.7 ± 16.2^**^	59.4 ± 3.6^**^	24.62 ± 1.12^**^	12.31 ± 2.10^**^	7.41 ± 0.07^**^	3.59 ± 0.09 ^ns^	2.15 ± 0.08^**^
T_2_	WT	7.0 ± 1.4	24.02 ± 0.64	133.6 ± 9.2	85.0 ± 2.1	27.98 ± 0.88	26.32 ± 6.25	7.45 ± 0.11	3.70 ± 0.02	2.28 ± 0.07
	*sd1-wx-1*	7.4 ± 1.4 ^ns^	23.59 ± 1.25 ^ns^	133.2 ± 16.4 ^ns^	80.8 ± 3.9 ^ns^	27.35 ± 1.08 ^ns^	26.78 ± 2.79 ^ns^	7.48 ± 0.19 ^ns^	3.69 ± 0.06 ^ns^	2.23 ± 0.09^**^
	*sd1-1*	7.2 ± 1.9 ^ns^	22.22 ± 0.84^**^	134.8 ± 18.5 ^ns^	86.6 ± 4.7 ^ns^	25.27 ± 0.92^**^	24.08 ± 3.11 ^ns^	7.36 ± 0.08^*^	3.52 ± 0.08^**^	2.16 ± 0.07^**^
	*sd1-2*	6.7 ± 0.9 ^ns^	23.31 ± 0.24^**^	133.2 ± 9.4 ^ns^	74.8 ± 5.6^**^	25.84 ± 0.86^**^	22.95 ± 3.70 ^ns^	7.55 ± 0.11^*^	3.66 ± 0.04^*^	2.22 ± 0.07^**^
	*sd1-3*	6.8 ± 1.0 ^ns^	22.37 ± 0.83^**^	96.8 ± 10.6^**^	69.2 ± 3.4^**^	25.14 ± 0.95^**^	16.5 ± 2.47^**^	7.60 ± 0.09^**^	3.62 ± 0.05^**^	2.19 ± 0.08^**^
T_3_	WT	8.4 ± 0.5	28.50 ± 0.69	142.9 ± 13.5	78.9 ± 2.8	26.66 ± 0.74	31.97 ± 3.47	7.40 ± 0.09	3.77 ± 0.03	2.25 ± 0.07
	*sd1-wx-1*	9.0 ± 1.1 ^ns^	25.18 ± 1.14^**^	136.1 ± 11.3 ^ns^	75.6 ± 2.6 ^ns^	26.12 ± 0.85 ^ns^	31.83 ± 5.25 ^ns^	7.40 ± 0.08 ^ns^	3.78 ± 0.05 ^ns^	2.23 ± 0.08 ^ns^
	*sd1-1*	9.1 ± 1.6 ^ns^	24.08 ± 1.09^**^	125.1 ± 10.7^*^	70.0 ± 2.6^**^	25.57 ± 0.32^**^	29.27 ± 5.72 ^ns^	7.38 ± 0.07 ^ns^	3.73 ± 0.05^*^	2.09 ± 0.07^**^
	*sd1-2*	9.3 ± 1.1 ^ns^	24.36 ± 1.16^**^	123.7 ± 13.8^*^	64.1 ± 1.2^**^	25.78 ± 0.39^**^	29.50 ± 1.23 ^ns^	7.55 ± 0.10^**^	3.79 ± 0.08 ^ns^	2.20 ± 0.05^**^
	*sd1-3*	9.0 ± 0.7 ^ns^	23.62 ± 0.82^**^	87.4 ± 11.8^**^	61.3 ± 4.9^**^	25.67 ± 0.33^**^	20.37 ± 4.34^**^	7.79 ± 0.10^**^	3.76 ± 0.05 ^ns^	2.18 ± 0.09^**^

The data are the means of at least five duplicates. WT means wild-type, PN means panicle numbers, PL means panicle length, GNPP means grain number per panicle, SSR means seed setting rate, 1000-GW means 1000-grain weight, YPP means yield per plant, GL means grain length. GW means grain width and GT means grain thickness. ^*^ represent the significant difference p < 0.05, ^ns^ and ^**^ represent the non-significant difference and significant difference at p < 0.01.

### Transcriptomic analysis in *sd1*-*wx* double mutants

**Figure 5:**
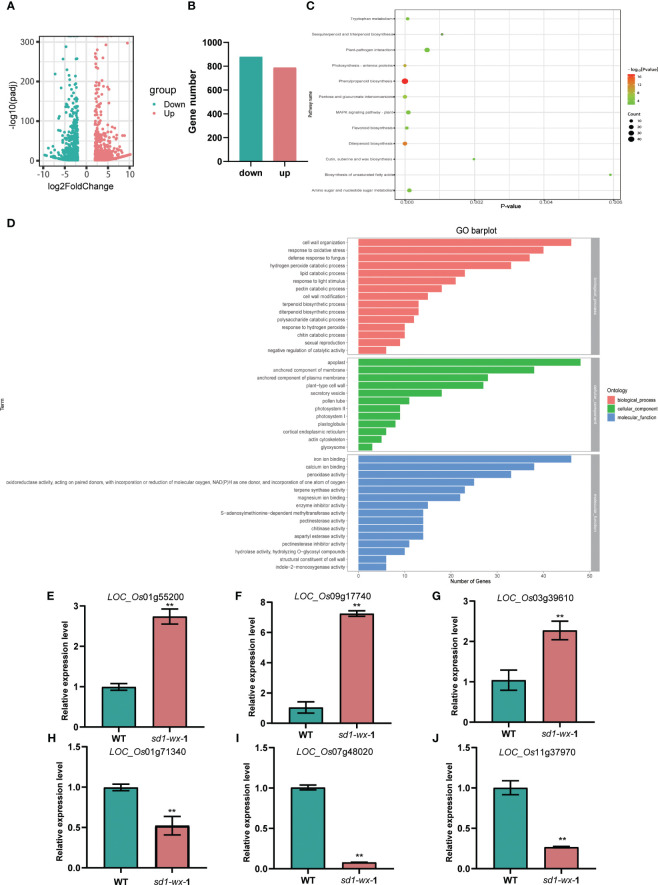
RNA-seq analysis between the *sd1-wx* and WT. **(A)** Volcano plot displaying DEGs between the *sd1-wx* and WT. **(B)** The number of DEGs in *sd1-wx* double mutant lines. **(C)** KEGG classifications of DEGs. Enrichment 12 significant pathways (both *p* value and *p* adjust value <0.05). **(D)** GO analysis of the DEGs. Enriched significant different GO terms with both *p* value and *p* adjust value<0.05. **(E-J)** RT-qPCR to confirm the transcript level of down-regulated genes *LOC_Os01g71340* (*PR2*), *LOC_Os07g48020* (*POX22.3*), and *LOC_Os11g37970* (*PR4a*) and up-regulated genes *LOC_Os01g55200* (*KAT1*), *LOC_Os09g17740* (*LHCB1.3*), and *LOC_Os03g39610* (*LHCB2*) at the stem.

To evaluate the impact of the *sd1*-*wx* knockout double mutant line on the rice transcriptome, we employed RNA-seq analysis on both *sd1-wx* double mutant and WT plants at the heading stage. Comparison of the transcriptomes of WT and *sd1-wx* mutant stems during the heading stage revealed 1665 DEGs, with 787 downregulated and 878 upregulated genes (**Figures 5A, B**; **Supplementary Table S3**). Employing GOseq30 for functional categorization, the DEGs were classified into 76 subclasses (**Supplementary Table S5**). The enriched GO terms are predominantly related to molecular functions and biological processes. Notably, the DEGs were significantly involved in biological processes and cellular components, with 54 secondary items exhibiting substantial enrichment (*p* value < 0.01 and *p* adjust value < 0.01). Among these, the top five items were apoplast (gene ratio 3.96%, GO:0048046), anchored component of membrane (gene ratio 3.14%, GO: 0031225), hydrogen peroxide catabolic process (gene ratio 2.72%, GO:0042744), peroxidase activity (gene ratio 2.72%, GO:0004601), and terpene synthase activity (gene ratio 1.90%, GO:0010333).

**Figure 6:**
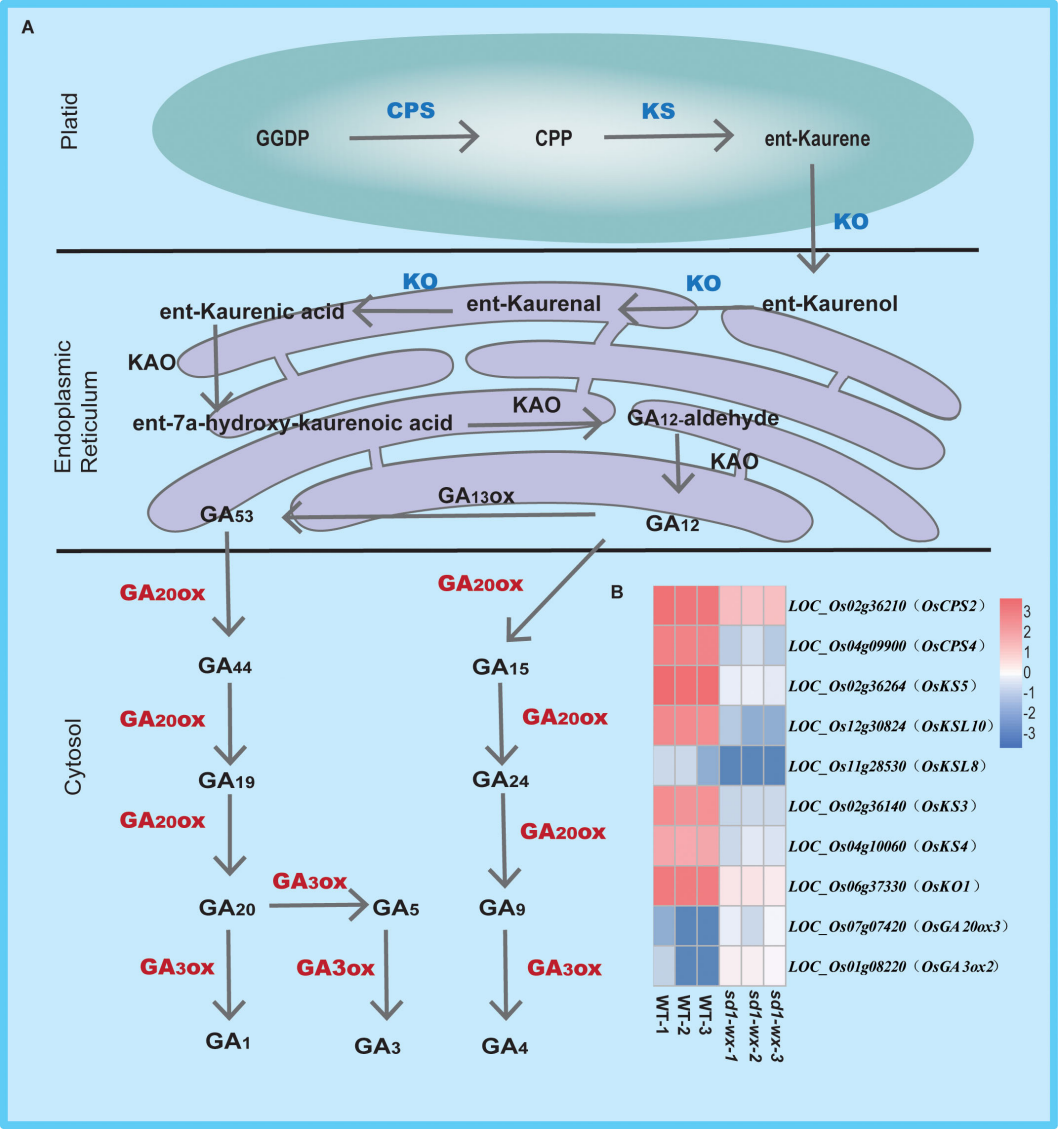
Pathway of gibberellin biosynthesis in WT and *sd1* knockout plants. **(A)** Pathway of gibberellin biosynthesis. **(B)** Heatmap analysis of the expression of 10 DEGs associated with gibberellin biosynthesis. Note: red indicates up-regulated genes, blue indicates down-regulated genes.

To further understand the biological function, we delving into biological pathways via KEGG pathways. 194 out of the 1665 DEGs were implicated in 12 pathways (**Supplementary Table S4**). The top two enriched pathways were phenylpropanoid biosynthesis (ko00940, gene ratio 10.50%) and diterpenoid biosynthesis (ko00904, gene ratio 4.11%), aligning with the results obtained from GO analysis. These pathways included annotations in peroxidase activity and terpene synthase activity. Additionally, other pathways such as photosynthesis-antenna proteins, pentose and glucuronate interconversions, flavonoid biosynthesis, tryptophan metabolism, MAPK signaling pathway-plant, amino sugar and nucleotide sugar metabolism, plant-pathogen interaction, sesquiterpenoid and triterpenoid biosynthesis, cutin, suberine and wax biosynthesis, and biosynthesis of unsaturated fatty acids were identified (**Figure 5C**; **Supplementary Table S4**). Furthermore, we found that most of the DEGs related to diterpenoid biosynthesis were down-regulated in *sd1*-*wx* double mutants. There were 18 genes associated with diterpenoid biosynthesis, including two gene encoding ent-copalyl diphosphate synthase (*OsCPS2*, *OsCPS4*), which is the first key enzyme catalyzes gibberellin biosynthesis pathway, five genes encoding terpene synthase/ent-kaurene synthase (*OsKS3*, *OsKS4, OsKS5, OsKSL8, OsKSL10*), which is the second enzyme in the gibberellin synthesis step, one gene encoding ent-kaurene oxidase (*OsKO1*), six genes encoding cytochrome P450, two genes encoding short-chainalcohol dehydrogenase (*OsSDR110C-MS1*, *OsSDR110C-MS2*), two gene encoding gibberellin biosynthesis gene (*OsGA20ox3*, *OsGA3ox2*) (**Figure 6**; **Supplementary Table S2**). This comprehensive RNA-seq analysis at the stem level unequivocally confirms the influence of *sd1* knockout on the expression of gibberellin biosynthesis-related genes.

To validate the DEGs identified from the RNA-seq analysis, we used RT-qPCR to determine the expression levels of the DEGs. We randomly selected 6 genes that were significantly up-regulated or down-regulated compared to WT. The results showed that the expression levels of three randomly selected up-regulated DEGs, *LOC_Os01g55200* (*KAT1*), *LOC_Os09g17740* (*LHCB1.3*), and *LOC_Os03g39610* (*LHCB2*) RT-qPCR results were greatly reduced, while three randomly selected down-regulated DEGs, *LOC_Os01g71340* (*PR2*), *LOC_Os07g48020* (*POX22.3*), and *LOC_Os11g37970* (*PR4a*) RT-qPCR results were greatly increased (**Figures 5E-J**). The RT-qPCR results were in good agreement with the RNA-seq analysis.

## Discussion

The quality of rice and its resistance to lodging are pivotal factors influencing both grain quality and yield potential. These traits are primarily governed by the major genes *Wx* and *SD1*, respectively. Using CRISPR/Cas9 genome editing technology for crop improvement has demonstrated remarkable efficiency. In the present study, we not only employed this technology to generate *Wx* gene or *SD1* gene knockout single mutant, but also utilized it to produce *sd1-wx* double mutants. Ultimately, this approach led to the development of elite rice lines with desirable traits, characterized by semi-dwarfism and glutinous properties. In addition, our investigation extended beyond genetic modifications to encompass a comprehensive analysis of various agronomic traits, quality attributes, physiological characteristics and physical properties of the modified plants. Furthermore, a comparative RNA-seq analysis was performed on both *sd1-wx* double mutants and WT plants. The integrative analysis of transcriptomic and physiological data of *sd1-wx* double mutants knockout lines could deepen our understanding of the development of plant height and amylose metabolism in rice.

In our current investigation, we have successfully generated an exceptional breeding line, denoted as XDW*^sd1^* ^-^ *^wx^* ^-1^, featuring double mutant plants. Notably, this edited line exhibited a reduction in endogenous gibberellin content, leading to a decrease in plant height, although it does not represent the absolute minimum height within the mutant lines, and a concomitant decline in amylose content. Interestingly, despite these alterations, agronomic traits, yield parameters, and seed germination rates demonstrated no statistically significant differences. Our findings align with previous reports highlighting the efficacy of CRISPR/Cas9 genome editing technology in manipulating the *SD1* gene to achieve lower plant height and enhanced yield potential (Han et al., 2019; Hu et al., 2019; Nawaz et al., 2020). Some researchers have suggested that although plant height decreases, seed yield remains unaffected due to the selective expression of *GA20ox-2* in young tissues, while its homologous gene, *GA20ox-1*, is preferentially expressed in unopened flowers (Sasaki et al., 2002). Our exploration further uncovered intriguing characteristics in the XDW*^sd1-^* ^3^ mutant line, characterized by the lowest endogenous gibberellin content and the most diminutive plant height among the mutants. Despite a lower seed setting rate, this mutant line exhibited an elongated grain length. The observed decrease in seed setting rate may be attributed to the absence of endogenous gibberellin content, potentially disrupting pollen development, as suggested by prior studies (Sakata et al., 2014).

Maintaining an optimal plant height is paramount for enhancing rice lodging resistance and overall yield. In our study, we engineered various alleles of the *SD1* gene, resulting in distinct plant height phenotypes. Specifically, the transgenic plants XDW*^sd1-2^* and XDW*^sd1^* ^-3^ harbored 1-bp and 23-bp nucleotide deletions in *SD1*, respectively. These deletions induced frame-shift mutations, predicting the translation of non-functional truncated proteins that disrupted gibberellin function. Consequently, these mutants exhibited the shortest plant height compared to the WT plants. Our observations extended to XDW*^sd1-wx^* ^-1^ and XDW*^sd1^* ^-1^ transgenic plants, carrying 3-bp and 18-bp nucleotide deletions at the first exon of the *SD1* gene. These deletions caused either one or six amino acid deletions in the *SD1* protein, respectively. Intriguingly, we also found that XDW*^sd1-wx^* ^-1^ and XDW*^sd1^* ^-1^ transgenic plants displayed varying degrees of endogenous gibberellin content and plant height reduction compared to the WT plants. The *SD1* gene encodes gibberellin 20 oxidase 2, a pivotal enzyme in gibberellin biosynthesis. However, despite its crucial role, the *SD1* gene did not emerge as differentially expressed in the comparative transcriptome analysis between *sd1-wx-*1 and WT plants. This anomaly suggests that the deletion of one or six amino acids might disrupt the catalytic activity of gibberellin 20 oxidase 2, thereby impairing the gibberellin metabolic pathway.

Gibberellins, a class of diterpenoids, are widely distributed in plants and are primarily synthesized in the young apical parts of plants (Hedden, 2020). The synthesis of gibberellins begins with the precursor geranylgeranyl diphosphate (GGPP), which undergoes sequential catalysis by ent-copalyl diphosphate synthase (CPS), ent-kaurenoic acid oxidase (KAO), ent-kaurene oxidase (KO), and ent-kaurene synthase (KS) to culminate in the formation of GA_12_, an integral intermediate in the gibberellin biosynthetic pathway (Hedden and Thomas, 2012; Hedden and Sponsel, 2015; Salazar-Cerezo et al., 2018; Hedden, 2020). Our study, utilizing comparative transcriptome analysis, unveiled a significant enrichment and down-regulation of differentially expressed genes associated with diterpenoid biosynthesis. Particularly noteworthy were the findings that two genes encoding CPS, five genes encoding KS, and one gene encoding KO were substantially down-regulated in *sd1-wx* double mutants. This observation raises the possibility that the compromised catalytic activity of gibberellin 20 oxidase 2 restricts the efficient conversion of GGPP to GA_12_ (**Figure 6**). Interestingly, we also found that two genes encoding gibberellin oxidase (*OsGA20ox3*, *OsGA3ox2*) exhibited remarkable up-regulated expression patterns in *sd1*-*wx* double mutants. This increased expression of gibberellin oxidase genes suggests a potential compensatory mechanism in response to the compromised function of gibberellin 20 oxidase 2. The up-regulation of these genes might serve to augment the production of downstream gibberellin compounds, offsetting the limitations imposed by the impairment in GA_12_ biosynthesis.

In our transcriptome analysis, we unearthed compelling insights into the regulatory landscape of gene expression. Phenylpropanoid biosynthesis emerged as the most significantly enriched pathway in the KEGG analysis, leading to 46 DEGs. Furthermore, the hydrogen peroxide catabolic process (enriched with 33 genes) and peroxidase activity (also enriched with 33 genes) could act as the most prominently enriched terms in the GO analysis (**Figure 5D**; **Supplementary Table S5**). These findings underscore a pronounced modulation of the phenylpropanoid metabolism machinery in *sd1-wx* double mutants. Noteworthy is the absence of down-regulation of the *Wx* gene expression in *sd1-wx* double mutants compared to the WT plants, as revealed by our transcriptome analysis. Equally intriguing is the lack of enrichment for genes related to the starch metabolism pathway. This discrepancy can be attributed to the specific high expression of *Wx* genes in the endosperm, contrasting with their low expression in the stem, a dynamic that underscores the intricacies of gene expression patterns in different plant tissues.

In conclusion, our study harnessed the precision of the CRISPR/Cas9 gene editing system to modify both the *SD1* and *Wx* genes in Xiangdaowan (XDW) rice, an elite landrace known for its robust aromatic properties. Across various generations, we successfully generated genetically stable, semi-dwarf glutinous rice lines, ensuring homozygosity. These edited lines exhibited a spectrum of alterations, including reduced gibberellin content, diminished plant height, lower AC, elevated GC, modified starch gelatinization characteristics in urea solutions, and the development of small holes in the endosperm microstructure. Notably, the germination rate and other key agronomic traits remained unaffected. To compensate for the growth defects associated with endogenous gibberellin deficiency, we applied exogenous GA_3_, revealing its potential to mitigate the observed growth-related issues. Our comparative transcriptome analysis uncovered that the compromised catalytic activity of gibberellin 20 oxidase 2 played a pivotal role in limiting the metabolism of diterpenoid biosynthesis. Taken together, our results highlight the efficacy of CRISPR/Cas9 technology in enhancing both plant height and grain quality in rice. By strategically manipulating key genes, we achieved improvements that not only offer agronomic benefits but also hold the potential to increase overall yield and enhance the quality of the crop.

## Data availability statement

The original contributions presented in the study are included in the article/**Supplementary Material**. Further inquiries can be directed to the corresponding author.

## Author contributions

QW: Conceptualization, Data curation, Formal analysis, Funding acquisition, Project administration, Resources, Validation, Visualization, Writing – original draft, Writing – review & editing. HG: Data curation, Formal analysis, Investigation, Methodology, Software, Writing – review & editing. KLi: Data curation, Formal analysis, Investigation, Methodology, Software, Writing – review & editing. HW: Data curation, Formal analysis, Methodology, Software, Writing – review & editing. FZ: Data curation, Formal analysis, Investigation, Software, Writing – review & editing. LW: Formal analysis, Investigation, Methodology, Software, Writing – review & editing. KLu: Formal analysis, Investigation, Software, Writing – review & editing. ML: Formal analysis, Software, Writing – review & editing. YS: Formal analysis, Software, Writing – review & editing. JZ: Funding acquisition, Validation, Writing – review & editing. WZ: Funding acquisition, Validation, Writing – review & editing. BP: Funding acquisition, Validation, Writing – review & editing. HY: Conceptualization, Project administration, Resources, Supervision, Validation, Writing – original draft, Writing – review & editing.
