# Supplementary material for: CRISPR/Cas9-mediated enhancement of semi-dwarf glutinous traits in elite Xiangdaowan rice (Oryza sativa L.): targeting SD1 and Wx genes for yield and quality improvement
Source: Front Plant Sci. 2024 Feb 16;15:1333191. doi: 10.3389/fpls.2024.1333191 (PMC10904601; doi:10.3389/fpls.2024.1333191)
Supplement: Supplementary file 1 [file Table_1.docx]

**Table S1 Primers used in this study**

| Primer name | Primer sequence(5'-3') |
| --- | --- |
| **Primers for cloning** | |
| sg RNA for *SD1* | GAGCCATTCGTGTGGCCGAACGG |
| sg RNA for *Wx* | GCTTGAGGCCCTGGAACCCGTGG |
| **Primers for quantitative RT-PCR** | |
| RT-*LHCB1.3*-F | TCTCCTCCCCGGTGATGGC |
| RT-*LHCB1.3*-R | GCGGTCTTGCGCATGGTGA |
| RT-*LHCB2*-F | ATGGGATTCGTCGAGGGCTAC |
| RT-*LHCB2*-R | TCGGCGAAGGTGTCAGGGTC |
| RT-*KAT1*-F | ACATATGCCTCCGGTACA |
| RT-*KAT1*-R | ACGCCTAAGCCTGCTGGTT |
| RT-*PR2*-F | ACATCGCCGTCGGCAACGAG |
| RT-*PR2*-R | GGTTCTCGTTGAACATGGCG |
| RT- *POX22.3* -F | CAGCTGCTCCAAGGTGAACTC |
| RT- *POX22.3* -R | AGATTTGCTTCCAGCAACGAA |
| RT- *PR4a* -F | CAACTGGGACCTGAACAAAGTG |
| RT- *PR4a*-R | GCGCCAAGACAACGGTTT |
| *UBIQ*-F | AACCAGCTGAGGCCCAAGA |
| *UBIQ*-F | ACGATTGATTTAACCAGTCCATGA |

**Table S2 Identified DEGs involved in** **gibberellin biosynthesis in WT and *sd1* knockout plants**

| Locus | Log2 fold change | Annotation | Synonym |
| --- | --- | --- | --- |
| LOC_Os02g36210 | -2.99^**^ | Ent-copalyl diphosphate synthase 2 | *OsCPS2/OsCyc2* |
| LOC_Os04g09900 | -6.48^**^ | Ent-kaurene synthase, chloroplast precursor, putative, expressed | *OsCPS4/OsCyc1* |
| LOC_Os02g36140 | -5.53^**^ | Terpene synthase, putative, expressed | *OsKS3/ OsDTC1* |
| LOC_Os04g10060 | -3.79^**^ | Ent-kaurene synthase, chloroplast precursor, putative, expressed | *OsKS4/ OsKSL4/ OsDTS2* |
| LOC_Os02g36264 | -5.85^**^ | Terpene synthase, putative, expressed | *OsKS5/OsKS6* |
| LOC_Os11g28530 | -4.62^**^ | Terpene synthase, putative, expressed | *OsKSL8/OsDTC2* |
| LOC_Os12g30824 | -7.76^**^ | Terpene synthase, putative, expressed | *OsKSL10* |
| LOC_Os06g37330 | -4.06^**^ | Ent-kaurene oxidase | *OsKO1* |
| LOC_Os04g10010 | -4.49^**^ | Short-chain alcohol dehydrogenase | *OsSDR110C-MS1* |
| LOC_Os04g10000 | -4.16^**^ | Short-chain alcohol dehydrogenase | *OsSDR110C-MS2* |
| LOC_Os01g08220 | 4.43^**^ | Gibberellin 3-beta-dioxygenase 2-2, putative, expressed | *OsGA3ox2* |
| LOC_Os07g07420 | 5.96^**^ | Gibberellin 20 oxidase 1-B, putative, expressed | *OsGA20ox3* |

^**^ represent the significant difference *p* < 0.01, ^*^ represent the significant difference *p* < 0.05
