# Supplementary material for: CRISPR/Cas9-mediated enhancement of semi-dwarf glutinous traits in elite Xiangdaowan rice (Oryza sativa L.): targeting SD1 and Wx genes for yield and quality improvement
Source: Front Plant Sci. 2024 Feb 16;15:1333191. doi: 10.3389/fpls.2024.1333191 (PMC10904601; doi:10.3389/fpls.2024.1333191)
Supplement: Supplementary file 5 [file Image_1.pdf]

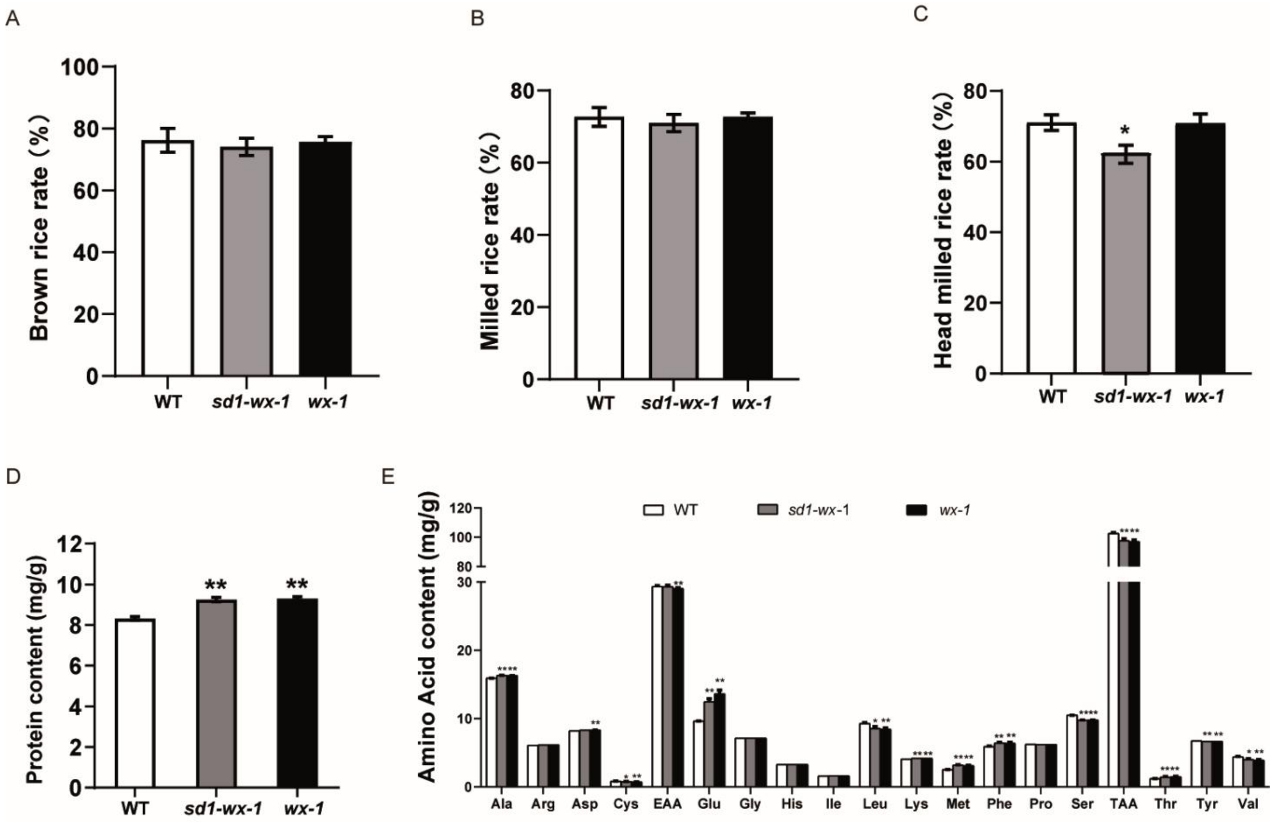

Fig S1. Processing quality and nutritional quality among WT and *sd1-wx* and *wx* mutants. Comparative analysis of brown rice rate (A) and milled rice rate (B) and head milled rice rate (C) among WT and *sd1*-*wx*-1 and *wx*-1. Applications of near infrared spectroscopy (NIRS) to evaluate protein content (D) and amino acid content (E) in WT and *sd1*-*wx*-1 and *wx*-1 seed.
